# Effects of dual arterial blood supply on liver regeneration in the graft and the host following heterotopic auxiliary liver transplantation

**DOI:** 10.3892/etm.2014.1976

**Published:** 2014-09-17

**Authors:** JUNJING ZHANG, JUNQING XI, CHAOXUAN DONG, XINGKAI MENG

**Affiliations:** 1 Department of General Surgery, Affiliated Hospital of Inner Mongolia Medical University, Huhhot, Inner Mongolia 010050, P.R. China; 2 Department of Pediatrics, University of Tennessee Health Science Center, Memphis, TN 38163, USA

**Keywords:** auxiliary liver transplantation, dual arterial blood supply, liver regeneration, portal vein arterializations

## Abstract

This study aimed to investigate the effect of the dual arterial blood supply method used in auxiliary liver transplantation on the regeneration of grafted and host liver. A total of 72 male Sprague-Dawley rats were randomly assigned to three experimental groups, namely the 68% hepatectomy group (group A), the 68% hepatectomy with dual arterial blood supply group (group B) and the auxiliary liver transplantation with dual arterial blood supply group (group C). Group C was further divided into the host liver subgroup (group Ca) and the transplanted liver subgroup (group Cb). Six animals from each group were sacrificed at 1, 2 and 7 days after surgery. The calculation of the liver regeneration rate (LRR) was based on measuring liver weight. Liver function was assessed by measuring serum alanine aminotransferase (ALT) levels. Immunohistochemistry was employed to detect the expression of proliferating cell nuclear antigen (PCNA). Apoptotic changes in the grafts and host livers were evaluated using TUNEL staining. The LRR in each group exhibited a tendency to increase over time. At each time point, the LRR of transplanted livers in group C exhibited no significant difference from that of host livers in group C (P>0.05). The ALT levels for each group exhibited a time-dependent decreasing tendency. The ALT level in group C was significantly higher compared to that in groups A and B at each time point (P<0.05). The expression of PCNA in transplanted and host livers in group C was significantly lower compared to that in groups A and B at the same time point (P<0.001). Although the number of apoptotic cells in each group varied at different time points, there was no statistically significant difference (P>0.05). In auxiliary liver transplantation with the dual arterial blood supply method, the capacity of the liver regeneration in the grafts was similar to that of the host livers. Therefore, this technique may reduce the potential risk of graft liver atrophy caused by functional competition.

## Introduction

Dual arterial blood supply in the liver is the technique of using other arteries to completely or partially replace blood supply from the portal venous system. Thus, the liver receives blood supply from one substitute artery and the hepatic artery. This method has been classified into the portal vein arterialization methods. However, this concept differs from the intrahepatic arteriovenous fistula or single blood supply of the liver due to complex reasons, such as hepatic artery injury due to resection of hilar cholangiocarcinoma (1–3). Since dual hepatic arterial blood supply methods were first used in liver transplantation, they have been applied to patients such as orthotopic liver transplant recipients with extensive portal vein thrombosis and those who cannot receive liver transplantation in the short term due to acute liver failure. In addition, traditional methods have been proven unable to improve the prognosis of such patients.

Although the application of dual hepatic arterial blood supply methods in patients under the above mentioned conditions was not common practice, a limited number of cases reported a good prognosis, with an improvement in liver function and morphology after several years of follow-up, which could not be achieved by traditional treatment methods (3). Therefore, it was hypothesized that this novel method could be of value for regular use in other liver transplantation patients as well. Our previous study (4) indicated that the dual hepatic arterial blood supply method was able to maintain normal liver function in rat liver transplantation models.

Auxiliary liver transplantation is widely used in patients with acute liver failure or metabolic liver diseases. Auxiliary liver transplantation has several advantages in theory; however, it is associated with a high incidence of vascular complications and functional competition between the grafted and the host livers, restricting the clinical long-term prognosis of the patients receiving liver transplantation (5,6). Either the vascular complications or the functional competition in auxiliary liver transplantation are associated with the blood supply of the portal venous system. In cases with functional competition of the transplanted and host livers, the blood flow of the portal vein to the transplanted liver is reduced and liver function gradually declines. However, after limiting the blood flow of the portal vein to the host liver and reestablishing the blood flow to the transplanted liver, the function of the transplanted liver may be restored (5). Auxiliary liver transplantation using the dual hepatic arterial blood supply method ensures the integrity of blood supply to the host liver, provides adequate blood supply for the transplanted liver and more flexible implant locations.

The regeneration of the graft and host livers in auxiliary liver transplantation may be regulated by the human body and may also be affected by dual arterial blood supply, indicating that the regeneration of the two livers in auxiliary liver transplantation has specific characteristics, compared to liver regeneration in conventional partial hepatectomy. Therefore, this study aimed to investigate the effects of the dual arterial blood supply method on the regeneration of the transplanted as well as the host liver in auxiliary liver transplantation, which may lay a solid foundation for the clinical application of the dual arterial blood supply method in auxiliary liver transplantation.

## Materials and methods

### Animals

A total of 72 male healthy Sprague-Dawley rats (10 weeks old and weighing 300–330 g) were purchased from Beijing Vital River Laboratory Animal Technology Co., Ltd., Beijing, China. The animals were provided standard laboratory chow and water *ad libitum* and were allowed to acclimatize for 1 week. All animal procedures were approved by the Institutional Animal Care and Use Committee of Inner Mongolia Medical University and complied with the ethical standards described in the NIH Guide for Laboratory Animals.

### Preparation of animal models

The animals were divided into three groups, namely the 68% hepatectomy group (group A, n=18), the 68% hepatectomy plus dual arterial blood supply group (group B, n=18) and the auxiliary liver transplantation plus dual arterial blood supply group (group C, donor n=18 and recipient n=18). Group C was further divided into the host liver subgroup (group Ca) and the transplanted liver subgroup (group Cb).

The animal models in groups A and B were prepared based on methods previously described (4,7). The animal model in group C was established on the basis of an improvement of the methods described by Schleimer *et al* (8). In brief, the recipient underwent a 68% liver and right kidney resection. The portal vein of the grafted liver with an equal volume of the host liver was connected to the right renal artery of the recipient via a stent of 0.5 mm inner diameter. The inferior vena cava of the transplanted liver was connected to the right renal vein of the recipient with the cuff technique. The celiac trunk of the transplanted liver with an aortal patch was anastomosed end-to-side with the abdominal aorta of the recipient. Finally, the tube secured in the donor bile duct was placed into the purse-string suture at the duodenum and fixed.

### Tissue and serum collection

All the rats were weighed prior to any intervention. The resected livers were also weighed when preparing the animal models. Six animals from each group were randomly selected and sacrificed sacrifced by exsanguination under ether anesthesia at 1, 2 and 7 days after surgery. Blood samples (2 ml) were collected from the inferior vena cava immediately after death and centrifuged at 32,000 × g for 3 min. The serum samples were frozen at −70°C until further use. All the livers were collected, weighed and fixed in 4% paraformaldehyde for immunohistochemistry and detection of apoptosis.

### Determination of the liver regeneration rate (LRR)

LRR was calculated in the three experimental groups at different time points based on the formula provided by previous studies (9,10). The method for calculating LRR in each group is described by the following formula:

LRR=(LW at autopsy-estimated residual LW at the time of surgery)/(resected LW)×100%

In the preliminary study, 50 rats of the same strain and similar body weights were observed. The ratio of liver weight (LW) to body weight (liver/body weight ratio) was 3.1±0.34% [mean ± standard error of the mean (SEM)]. In this study, the theoretical weight of the whole liver may be estimated using the liver/body weight ratio and measured body weight. The estimated residual LW at the time of surgery was obtained by deducting the resected LW from the calculated whole LW. Resected LW was measured by weighting the resected left and middle liver lobes during the operation. LW at autopsy was obtained by measuring each liver after the animals were sacrificed.

### Detection of alanine aminotransferase (ALT)

The ALT concentrations in all the samples were detected using a automatic biochemical analyzer (Modular DPP System; Roche Modular DPP, Hitachi Ltd., Tokyo, Japan).

### Proliferating cell nuclear antigen (PCNA) expression detection

The expression of PCNA was detected using an immunohistochemical SABC assay with a mouse monoclonal antibody against PCNA (BM0104, Boster Biological Engineering Co, Ltd, Wuhan, China) (4). PCNA-positive cells exhibited brownish-yellow or yellow nuclei. If brown particles appeared in the nucleus, the cells were considered as positive. A total of 5 different high-power fields (magnification, ×100) were randomly selected on each section and the number of positive cells in each field was counted.

### Apoptosis test using TUNEL assay

Apoptosis was detected using the TUNEL assay as previously described (4) and brownish yellow nuclei were considered to be positive. One section was randomly obtained from each sample in each group. A total of 5 fields were selected at high magnification (x400) and the number of total cells and TUNEL-positive cells was counted.

### Statistical methods

All the data are expressed as means ± SEM and analyzed using SPSS statistical software, version 13.0 (SPSS Inc., Chicago, IL, USA). The F test was used to determine homogeneity of variance and all the groups at each observation time point were compared using the t-test. P<0.05 was considered to indicate a statistically significant difference.

## Results

### LRR

**Figure 1:**
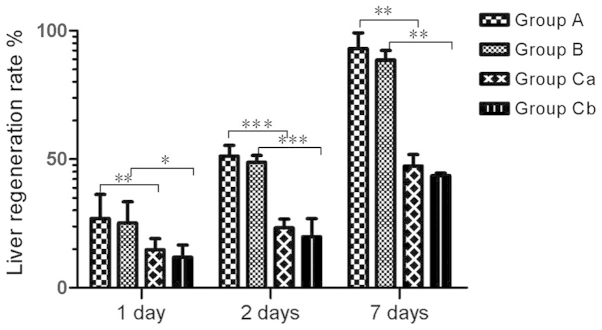
Liver regeneration rate of liver tissue at different time points after surgery. ^*^P<0.05, ^**^P<0.01, ^***^P<0.001. Group A, 68% hepatectomy; group B, 68% hepatectomy plus dual arterial blood supply; group Ca, auxiliary liver transplantation plus dual arterial blood supply, host liver; and group Cb, auxiliary liver transplantation plus dual arterial blood supply, graft liver.

The LRR in all the experimental groups exhibited a tendency to increase over time, from 26.95±9.34% (group A), 25.2±8.27% (group B), 19.72±9.30% (group Ca) and 11.9±4.75% (group Cb) at surgery to 93.00±6.13% (group A), 88.65±3.64% (group B), 47.40±4.40% (group Ca) and 43.60±1.03% (group Cb) (Fig. 1). At 1 and 7 days after surgery, there was no significant difference in the LRR between the grafted and the host liver in group C (P>0.05). During the observation period, the LRR of the host and grafted livers in group C decreased significantly compared to that in groups A and B (Fig. 1).

### ALT levels

**Figure 2:**
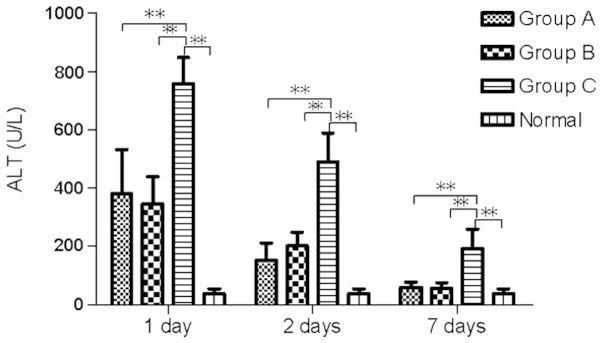
Alanine aminotransferase (ALT) levels in each group at different time points after surgery. ^**^P<0.01. Group A, 68% hepatectomy; group B, 68% hepatectomy plus dual arterial blood supply; group Ca, auxiliary liver transplantation plus dual arterial blood supply, host liver; and group Cb, auxiliary liver transplantation plus dual arterial blood supply, graft liver.

The ALT levels in each group decreased gradually in a time-dependent manner. At day 7 after surgery, the ALT levels of groups A and B approached normal values, whereas the ALT concentrations in group C remained significantly higher than normal. At each time point, the ALT levels in group C were significantly higher compared to those in groups A and B (Fig. 2).

### PCNA expression in liver tissue

**Figure 3:**
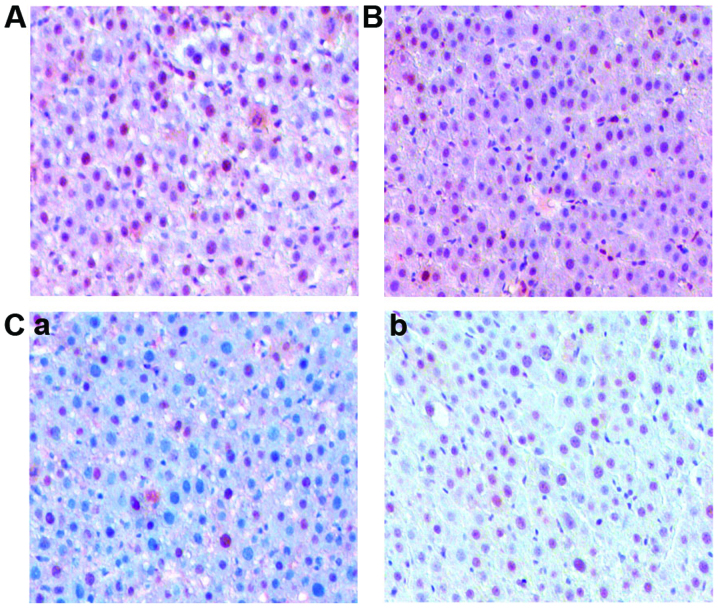
PCNA expression in liver tissue in each group at day 2 after surgery (magnification, ×100). (A) 68% hepatectomy; (B) 68% hepatectomy plus dual arterial blood supply; (Ca) auxiliary liver transplantation plus dual arterial blood supply, host liver; and (Cb) auxiliary liver transplantation plus dual arterial blood supply, graft liver.

**Figure 4:**
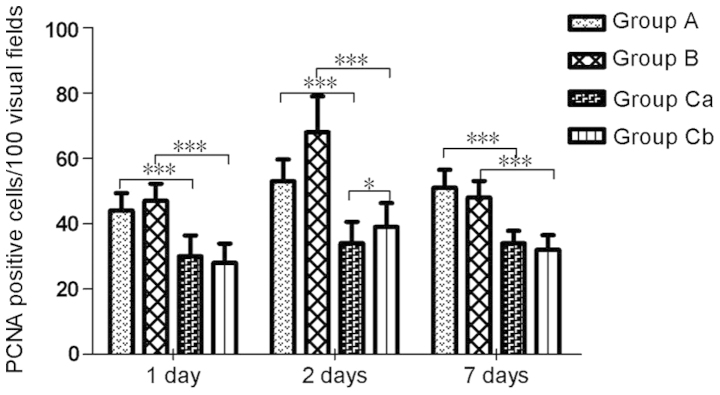
Level of proliferating cell nuclear antigen (PCNA) expression in each group at different time points after surgery. ^*^P<0.05, ^***^P<0.001. Group A, 68% hepatectomy; group B, 68% hepatectomy plus dual arterial blood supply; group Ca, auxiliary liver transplantation plus dual arterial blood supply, host liver; and group Cb, auxiliary liver transplantation plus dual arterial blood supply, graft liver.

The immunohistochemical staining images at day 2 after surgery are presented in Fig. 3. At each time point, the expression of PCNA in group C was significantly lower compared to that in groups A and B (P<0.001) (Fig. 4). At day 2 after surgery, the expression of PCNA in group Cb was significantly higher compared to that in group Ca (P<0.05) (Fig. 4).

### Apoptosis detection

**Figure 5:**
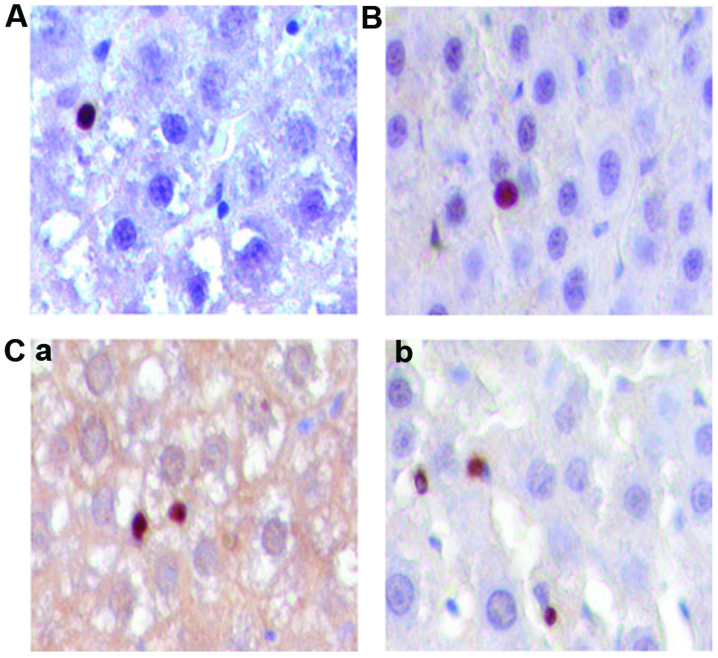
Expression of apoptotic cells in each group at day 7 after surgery (magnification, ×400). (A) 68% hepatectomy; (B) 68% hepatectomy plus dual arterial blood supply; (Ca) auxiliary liver transplantation plus dual arterial blood supply, host liver; and (Cb) auxiliary liver transplantation plus dual arterial blood supply, graft liver.

**Figure 6:**
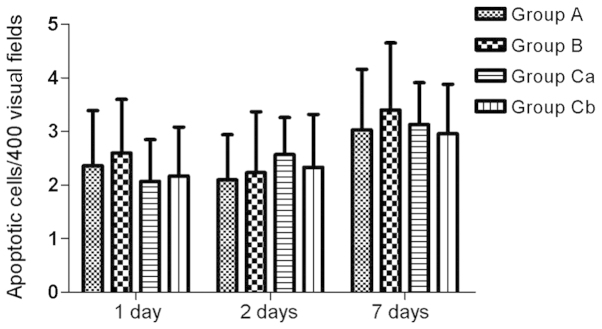
Level of apoptotic cells in each group at different time points after surgery. Group A, 68% hepatectomy; group B, 68% hepatectomy plus dual arterial blood supply; group Ca, auxiliary liver transplantation plus dual arterial blood supply, host liver; and group Cb, auxiliary liver transplantation plus dual arterial blood supply, graft liver.

Typical images of liver cell apoptosis detected using the TUNEL assay are presented in Fig. 5. There were no statistically significant differences in the number of apoptotic cells among groups at each time point (P>0.05) (Fig. 6). The number of apoptotic cells in groups A and B decreased within 24 h after surgery and then increased. In group C, the number of apoptotic cells in the host liver and the transplanted liver steadily increased after surgery (Fig. 6).

## Discussion

In auxiliary liver transplantation using the dual arterial blood supply method, the abdominal cavity accommodated the donor as well as the recipient livers and the grafted liver received a dual arterial blood supply, resulting in changes in the anatomical structure and blood supply in the grafted and host livers. Based on these changes, the questions of whether the regeneration in the grafted and host livers in auxiliary liver transplantation is distinguished from that in conventional partial hepatectomy and of whether the interaction between the donor and host livers leads to the regenerative changes were raised (3). It was previously demonstrated that the arterialized blood flow in 68% hepatectomy with dual hepatic arterial blood supply (a stent of 0.5 mm inner diameter was used to connect the right renal artery and portal vein) was similar to the normal physiological flow of the portal vein (10). In our previous study, we also observed that the LRR of rats receiving 68% hepatectomy with dual arterial blood supply was similar to that of the rats receiving simple 68% hepatectomy (4).

Schleimer *et al* (11) established the model of auxiliary liver transplantation with the dual arterial blood supply method using a stent of 0.3 mm inner diameter, but observed a functional competition between the transplanted and recipient livers and gradual shrinking of the graft, due to the lack of the hepatotrophic factor of the portal venous system. However, another relevant study (12) demonstrated that the amount of the residual host liver affected the weight of the transplanted liver, in the absence of the transplanted liver in rats receiving portal venous blood supply. When 30% of the host liver was resected, obvious hyperplasia of the transplanted liver was observed. Recently, Ringers *et al* (13) reported that the portal vein in the transplanted liver, after receiving blood supply from the left renal vein, could ensure the long-term normal function of the liver transplant. Based on the above mentioned findings and the results of our previous study (4), we infer that the long-term function of the transplanted liver in auxiliary liver transplantation does not depend on the blood supply from the portal venous system, but rather on adequate portal blood flow, regardless of it being arterial or venous blood. In our established animal models with auxiliary liver transplantation using the dual arterial blood supply method (with a stent of 0.5 mm inner diameter), the LRR of the transplanted and host livers during the observation period was significantly lower compared to that in the 68% liver resection animal models; however, there was no difference in liver regeneration between the grafted and the host livers. This finding confirmed that adequate blood flow amount in the portal vein determines long-term liver function following liver transplantation. The results of this study differed from those of Schleimer *et al* (11). The differences may be attributed to the diameter of the stent placed to ensure adequate portal blood flow. In this study, the observation period was 7 days, which was based on the complete regeneration time of the liver in classic partial hepatectomy. At 7 days following 68% liver resection, the liver mass of the rats recovered to 95–100% of the normal liver weight. We aim to prolong the observation time of this study to draw more definitive conclusions.

Sauvanet *et al* (14) reported that a whole or half transplanted liver to an ectopic site may promote regeneration of the host liver in their established rat model following acute liver failure induced by 80% liver resection treated by auxiliary liver transplantation. Even with the same surgical procedure, whole-liver transplants may more effectively promote the regeneration of the host liver in the short term compared to partial liver transplants. Following observation for 1 month, the total weight of the liver of the donor and the recipient in the whole-liver transplants group was significantly higher compared to the weight of standard liver required by the recipient. We previously established the mini-pig model of portal hypertension, with splenectomy plus auxiliary heterotopic partial liver transplantation. The implanted liver was able to maintain normal liver function (15). The total weight of the donor and the recipient livers was significantly higher compared to the weight of standard liver required by the recipient, which was similar to the findings of Sauvanet *et al* (14). However, other studies (11,16) demonstrated that the total weight of the liver required by the recipients following transplantation was regulated by body weight and the final ratio of liver weight to body weight was constant. In our established animal model with the auxiliary liver transplantation using the dual arterial blood supply method, the weights of the transplanted and host livers at day 7 after surgery were significantly lower compared to those of groups A and B, but the total weight of the transplanted and the host liver was similar to the original liver weight of the model, which was consistent with the findings in Bismuth *et al* (16) and Schleimer *et al* (11) studies.

In this study, we proposed the novel concept of ‘functional liver weight’, defined as the effective liver weight required to maintain normal liver function, which may be affected by anatomical as well as physiological factors. The ratio of ‘functional liver weight’ to body weight should be constant, indicating that an increase in liver weight is not necessarily accompanied by an increase in its physiological function. Under pathological conditions, such as liver cirrhosis, the required weight of the liver parenchyma increased to meet the body’s metabolic needs, while under normal liver function conditions, the required weight of the liver should be similar to the original weight of the normal liver.
